# Lipid phosphate phosphatase 3 regulates adipocyte sphingolipid synthesis, but not developmental adipogenesis or diet-induced obesity in mice

**DOI:** 10.1371/journal.pone.0198063

**Published:** 2018-06-11

**Authors:** Lorenzo Federico, Liping Yang, Jason Brandon, Manikandan Panchatcharam, Hongmei Ren, Paul Mueller, Manjula Sunkara, Diana Escalante-Alcalde, Andrew J. Morris, Susan S. Smyth

**Affiliations:** 1 Division of Cardiovascular Medicine, The Gill Heart and Vascular Institute, University of Kentucky, Lexington, KY, United States of America; 2 División de Neurociencias, Instituto de Fisiología Celular, Universidad Nacional Autónoma de México, Ciudad de México, CDMX, México; 3 Department of Veterans Affairs Medical Center, Lexington, Kentucky, United States of America; Virginia Commonwealth University Medical Center, UNITED STATES

## Abstract

Dephosphorylation of phosphatidic acid (PA) is the penultimate step in triglyceride synthesis. Adipocytes express soluble intracellular PA-specific phosphatases (Lipins) and broader specificity membrane-associated lipid phosphate phosphatases (LPPs) that can also dephosphorylate PA. Inactivation of lipin1 causes lipodystrophy in mice due to defective developmental adipogenesis. Triglyceride synthesis is diminished but not ablated by inactivation of lipin1 in differentiated adipocytes implicating other PA phosphatases in this process. To investigate the possible role of LPPs in adipocyte lipid metabolism and signaling we made mice with adipocyte-targeted inactivation of LPP3 encoded by the *Plpp3*(*Ppap2b*) gene. Adipocyte LPP3 deficiency resulted in blunted ceramide and sphingomyelin accumulation during diet-induced adipose tissue expansion, accumulation of the LPP3 substrate sphingosine 1- phosphate, and reduced expression of serine palmitoyl transferase. However, adiposity was unaffected by LPP3 deficiency on standard, high fat diet or Western diets, although Western diet-fed mice with adipocyte LPP3 deficiency exhibited improved glucose tolerance. Our results demonstrate functional compartmentalization of lipid phosphatase activity in adipocytes and identify an unexpected role for LPP3 in the regulation of diet-dependent sphingolipid synthesis that may impact on insulin signaling.

## Introduction

Diet-induced expansion of adipose tissue is the fundamental mechanism underlying the increasing incidence of obesity, with its associated co morbidities of diabetes, cardiovascular disease and cancer [1]. Phosphatidic acid (PA) is a common intermediate in the synthesis of triglycerides and phospholipids from dietary fats and carbohydrates [2]. Enzymatic dephosphorylation of PA generates diacylglycerol (DG) which is then acylated to produce triglycerides, and both PA and DG are substrates for enzymes that initiate synthesis of the major classes of phospholipids [3]. In mammals, the membrane-associated PA phosphatase activities are encoded by the *Plpp* genes (previously known as *Ppap2a*, *Ppap2c*, and *Ppap2b*) [4]. In addition to PA, these enzymes can dephosphorylate a broader range of lipid phosphate substrates including lysophosphatidic acid (LPA), sphingosine 1-phosphate (S1P) and ceramide 1-phosphate (C1P) which are intra- and extra-cellular bioactive mediators [5]. These membrane associated enzymes are termed lipid phosphate phosphatases (LPPs) to reflect this broader substrate selectivity. LPPs are localized to intracellular membrane compartments and to the plasma membrane. The transmembrane topology of these enzymes places the active site facing the lumen on intracellular membranes or the extracellular space[4]. While manipulations of LPP expression in cells and animals results in changes in intracellular lipid metabolism and signaling these enzymes are also implicated as cell surface regulators of signaling by bioactive LPA and S1P [6].

Mutations that inactivate the PA phosphatase lipin result in severe lipodystrophy in mice. Diet-dependent triglyceride synthesis is diminished, but not absent, in adult mice in which the lipin1 gene has been inactivated in post-differentiated adipocytes, implying that lipin1 functions redundantly with other PA phosphatases to sustain triglyceride synthesis in adipose tissue [7, 8]. The LPP enzymes appear to serve a non-redundant role [6, 9–13] and could function in adipose triglyceride synthesis. While more recent data suggests that the compensatory PA phosphatase activity that sustains triglyceride synthesis in the absence of lipin1 may involve the related lipin2 and lipin3 isoforms [9], adipocytes also express LPP3, encoded by the *Plpp3* gene [14]. Inactivation of *Plpp3* in mice results in early embryonic lethality due to defects in vasculogenesis and patterning [10]. Consequently, while mice with targeted inactivation of *Plpp3* in neuronal [15] and vascular [16, 17] tissues have been described nothing is presently known about the possible function of *Plpp3* and its product LPP3 in adipose tissue.

In addition to their possible contribution to intracellular triglyceride and lipid metabolism, determining the role of LPPs in adipocyte development and function is particularly interesting in light of their established ability to inactivate the extracellular bioactive lipids LPA and S1P. Extracellular LPA is generated by the lysophospholipase D activity of autotaxin (ATX) and elicits cellular responses via G-protein coupled receptors [18]. Differentiating adipocytes express ATX and can produce extracellular LPA in response to adrenergic stimulation [19]. Increases in ATX levels in db/db mice correlate with obesity, and plasma levels of ATX are reduced by adipocyte-specific inactivation of the *Enpp2* gene encoding ATX [20]. Data from both cell culture experiments and genetically manipulated mice support roles for ATX, LPA and the LPA1 receptor in adipose tissue development and indicate that these processes may be regulated by LPPs. For example, during preadipocyte differentiation in culture, LPP gene expression decreases concurrently with decreased metabolism of LPA and increased sensitivity to extracellular LPA [21]. LPP3, is expressed in both white and brown adipose tissue and alterations in LPP3 expression have been associated with defects in brown adipocyte precursor differentiation *in vitro* [22]. The patterning defects observed in LPP3 deficient mouse embryos have been attributed to effects on Wnt signaling [10] which is also known to regulate brown preadipocyte differentiation [23].

Taken together, the studies outlined above provide a strong rationale for investigating the potential role of LPP3 in adipose tissue development and function. To address these questions, we generated mice with adipocyte- specific inactivation of *Plpp3*. Our results indicate that LPP3 is dispensable for both lipid signaling pathways that regulate adipogenic differentiation and intracellular metabolic pathways necessary for dietary triglyceride synthesis. We report an unexpected role for LPP3 in the regulation of diet-induced sphingophospholipid synthesis, which was associated with improved glucose tolerance.

## Materials and methods

Unless noted otherwise, materials and reagents used in this study were from previously identified sources [6, 7, 16, 24].

### Animals

All procedures conformed to the recommendations of "Guide for the Care and Use of Laboratory Animals" (Department of Health, Education, and Welfare publication number NIH 78–23, 1996) and were approved by the Institutional Animal Care and Use Committee. Mice were weaned at 21 days, housed on a 14 h light and 10 h dark cycle, kept under standard humidity and temperature condition with free access to water and conventional rodent chow. C57BL/6 *Fabp4*-*Cre* (*AP2-Cre*) mice (Jackson Laboratory), which predominantly express the Cre recombinase in adipocytes, were crossed with C57BL/6 *Plpp3*^fl/fl^ mice with exons 3 and 4 flanked by *loxP* sites. At the time of weaning, mice were fed either a normal rodent diet (ND; 2018 Harlan Tekland Rodent Diet), high-fat diet (HFD) containing 60% fat, 20% protein, and 20% carbohydrate by calories (Research Diet D12492, New Brunswick, NJ), or Western diet (WD) containing 0.21% cholesterol (Research Diet D12079B). Body weights were recorded weekly and body composition was measured using dual-energy X-ray absorptiometry (DEXA; GE Lunar PIXImus software version 1.45; Lunar, Madison, WI) as previously described (27). Glucose and insulin tolerance tests were performed as previously described [24] after 8 weeks on the indicated diet. In brief, morning fasted mice were injected i.p. with glucose (2g/kg body weight) or insulin (Humalin at 0.5 U /kg) in isotonic saline and serial measurements of glucose level were taken from the tail tip before the injection and at 15, 30, 60 and 120 minutes after injection in conscious mice. Measurements of insulin, cytokines, and adipokines were performed on plasma as previously described (27). Animals were euthanized with carbon dioxide followed by cervical dislocation.

### Immunoblotting

Tissues were homogenized in lysis buffer (50 mmol/l Tris HCl, pH 6.8) containing 2.5% sodium dodecyl sulfate, 1% NP40, 10% glycerol, 10 mmol/l NaF, 0.1 mmol/l Na orthovanadate, and protease inhibitors using a stainless steel bead tissue homogenizer. Immunoblot analysis was performed with an anti-peptide polyclonal LPP3 antibody (custom generated by PolyScience), and visualized with the Licor Odyssey system (LI-COR, Lincoln, NE) using fluorescently conjugated secondary antibodies. Characterization of the antibody has been previously reported and its specificity for LPP3 has been demonstrated in tissue-specific knock-out mice (19).

### Quantitative RT-PCR measurements

Total RNA was extracted using Trizol reagent (Invitrogen, Carlsbad, CA) and quantitative, real-time, reverse- transcription PCR was conducted using a cDNA synthesis kit (Applied Biosystem, Carlsbad, CA) and gene- specific primers according to the manufacturer's directions. The threshold cycles (*C*_T_ value), corresponding to exponential amplification of PCR product during the log-linear phase for both the target genes and internal reference gene (Eukaryotic 18S rRNA Endogenous Control from Applied Biosystems), were analyzed for each sample in duplicate using the Δ*C*_T_ comparative method to determine relative expression levels. The relative quantification values (RQ) were calculated for each sample with the 2^-ΔΔCt^ formula. The reference sample was from wild-type animals.

### Mass spectrometry measurements of lipids

Lipids were measured using high performance liquid chromatography (HPLC) electrospray ionization (ESI) tandem mass spectrometry (MS) using AB Sciex 4000 Q-Trap linear ion trap triple quadrupole mass spectrometers or an AB Sciex 5600 Q-TOF quadrupole time of flight mass spectrometer and methods that have been reported in detail elsewhere [7, 16, 24–29]. In brief, these methods involve lipid extraction using acidified organic solvents with the inclusion of class specific lipids with mass labels or unnatural acyl chain lengths as internal or recovery standards. Quantitation was accomplished by selected ion monitoring mode HPLC MS/MS using calibration curves generated using independently quantitated standards [30, 31]. Data were normalized to tissue mass. Hexadecenal was quantitated as its semicarbazone derivative [29].

### Statistics

Results were expressed as mean ± SD and analyzed using unpaired one-tailed Student’s *t-* test or ANOVA, as indicated in the text or figure legends. Results were considered statistically significant at *P* < 0.05. Statistical analysis was performed using SigmaStat (San Jose, CA).

## Results

### LPP3 is dispensable for adipocyte development

**Fig 1:**
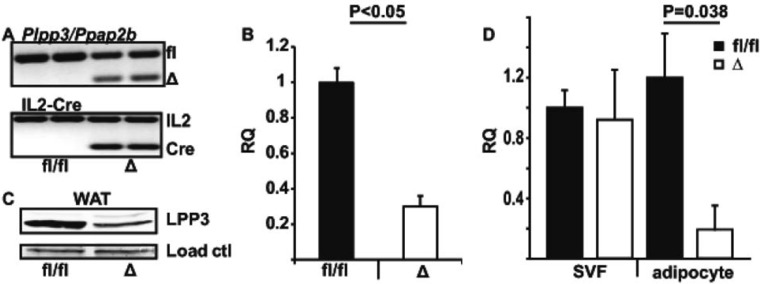
Adipose deletion of *Plpp3* effects on LPP3 mRNA and protein levels. A. PCR analysis of *Plpp3*^*fl/fl*^ (fl/fl) and AP2-Cre/*Plpp3*^Δ^ (Δ) mice. Top: specific primers were used to detect the floxed *Plpp3* allele (fl) and Cre recombinase-mediated deletion of exons 3 and 4 (Δ). Bottom: specific primers were used to detect the Cre transgene and IL2 expression as an internal control. The first two lanes are products from fl/fl animals and lanes 3 and 4 are from Δ mice. B. *Plpp3* gene expression measured by quantitative RT-PCR in white adipose tissue (WAT) from *Plpp3*^*fl/fl*^ (fl/fl) (black bars) and AP2-Cre/*Plpp3*^Δ^ (Δ) (open bars) mice (n = 3 per group). Values for mRNA expression are presented as relative quantification (RQ) normalized to a reference value obtained in a fl/fl animal and presented as mean ± SD. P<0.05 by Students t-test. C. Immunoblot analysis of LPP3 and a loading control in WAT from *Plpp3*^*fl/fl*^ (fl/fl) and AP2-Cre/*Plpp3*^Δ^ (Δ) mice. Equal amounts of total protein (were loaded 40 μg/lane) D. *Plpp3* gene expression measured by quantitative RT-PCR in stromal vascular fraction (SVF) or adipocytes from white adipose tissue from AP2-Cre/*Plpp3*^Δ^ (Δ) (dark bars) and *Plpp3*^*fl/fl*^ (fl/fl) (open bars) mice. Values for mRNA expression are presented as relative quantification (RQ) normalized to a reference value obtained in a fl/fl animal and presented as mean ± SD. (n = 3 / group).

To inactivate *Plpp3* expression in adipose tissue, we used previously reported mice in which exons 3 and 4 of *Plpp3* are flanked by loxP sites (fl/fl) [32]. These exons encode the second and third transmembrane domains, the first intracellular and the second outer loop, and 12 amino acids of the fourth transmembrane segment of LPP3. The Cre deleted *Plpp3* allele does not express detectable LPP3 protein [32]. Mice harboring the *Plpp3*-floxed allele (fl/fl) were bred with mice expressing Cre recombinase under the control of the *Fabp4* promoter (AP2-Cre). Cre recombinase-mediated deletion (Δ) of exons 3 and 4 of the *Plpp3* gene was verified by PCR (Fig 1A). Mating *Plpp3*^*fl/fl*^ with *Plpp3*^*fl/fl*^ mice carrying the AP2-Cre transgene resulted in transgene expression and exon excision in ~50% of the offspring at the time of weaning (21 days), indicating that tissue deletion of *Plpp3* driven by the *Fabp4* promoter did not result in embryonic or neonatal lethality. Quantitative PCR (Fig 1B) demonstrated substantially reduced *Plpp3* expression in white adipose from AP2-*Plpp3*^Δ^ (Δ) mice as compared to *Plpp3*^*fl/fl*^ mice (fl/fl). Similarly, immunoblot analysis (Fig 1C) of adipose tissue from *Plpp3*^*fl/fl*^ mice without or with AP2-Cre, indicated substantially lower levels of LPP3 expression in mice in which the floxed exons were deleted. Stromal cell [33] production of LPP3 may account for residual expression in these tissue preparations in the absence of adipose-derived LPP3 (Fig 1D).

**Fig 2:**
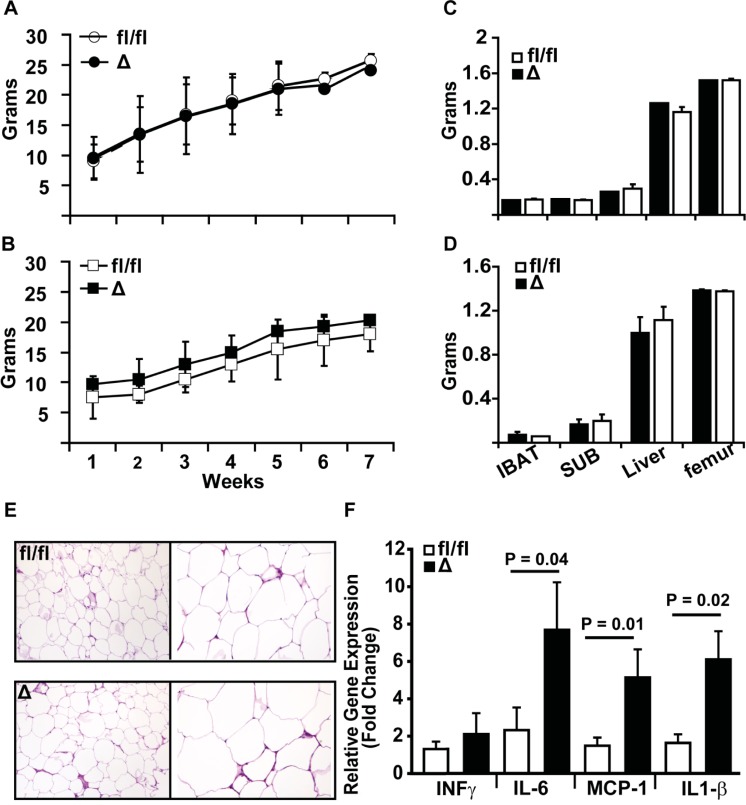
Deficiency of adipose LPP3 does not impact body size or adipose tissue development in mice on a standard diet. Body weight of male (A) and female (B) *Plpp3*^*fl/fl*^ (fl/fl; open symbols) and AP2-Cre/*Plpp3*^Δ^ (Δ; closed symbols) mice. Results are graphed as mean ± SD from 4–5 animals/group. Weight in grams of interscapular brown adipose tissue (IBAT), subcutaneous (SUB) white adipose tissue, and liver, and femur length (in mm) in 9-week old male (C) and female (D) *Plpp3*^*fl/fl*^ (fl/fl; open bars) and AP2-Cre/*Plpp3*^Δ^ (Δ; closed bars) mice. Comparisons between genotypes were made by Students t-test. E. Representative histology of *Plpp3*^*fl/fl*^ (fl/fl) and AP2-Cre/*Plpp3*^Δ^ (Δ) subcutaneous white adipose tissue stained with Periodic acid–Schiff (PAS) at 10x (left) and 20x (right) magnification. F. Expression analysis of inflammatory gene expression in visceral fat from *Plpp3*^*fl/fl*^ (fl/fl; open bars; n = 5) and AP2-Cre/*Plpp3*^Δ^ (Δ; closed bars; n = 4) mice on high fat diet. Results are graphed as mean ± SD and data analyzed by two-tailed t-test.

Mice lacking adipose LPP3 were phenotypically indistinguishable from their littermate controls. Both male (Fig 2A) and female (Fig 2B) AP2-Cre/*Plpp3*^Δ^ (Δ) mice displayed normal growth curves and body weights up to 12 weeks of age. The weight of interscapular brown adipose tissue (IBAT), subcutaneous (SUB) white adipose tissue, liver, and femur lengths were similar in *Plpp3*^*fl/fl*^ (fl/fl) and AP2-Cre/*Plpp3*^Δ^ (Δ) male (Fig 2C) and female mice (Fig 2D). When examined histologically, no differences were apparent in fat from mice lacking adipose LPP3 (Fig 2E). Together, these results indicate that adipose LPP3 is not required for normal development of brown or white fat in mice fed a standard diet. mRNA measurements revealed that expression of some genes encoding markers of inflammation including interleukin 6 (IL-6), monocyte chemotactic protein 1(MCP-1; CCL2), interleukin 1-Beta (IL-1β) but not all (INFγ) were higher in adipose from *Plpp3*^*fl/fl*^ (fl/fl) control mice in comparison with AP2-Cre/*Plpp3*^Δ^ (Δ), indicating a possible role in LPP3 in regulating obesity-stimulated inflammation (Fig 2F). Therefore, changes in lipid content were profiled.

### LPP3 deficiency blunts diet-induced sphingolipid accumulation in adipose tissue

**Fig 3:**
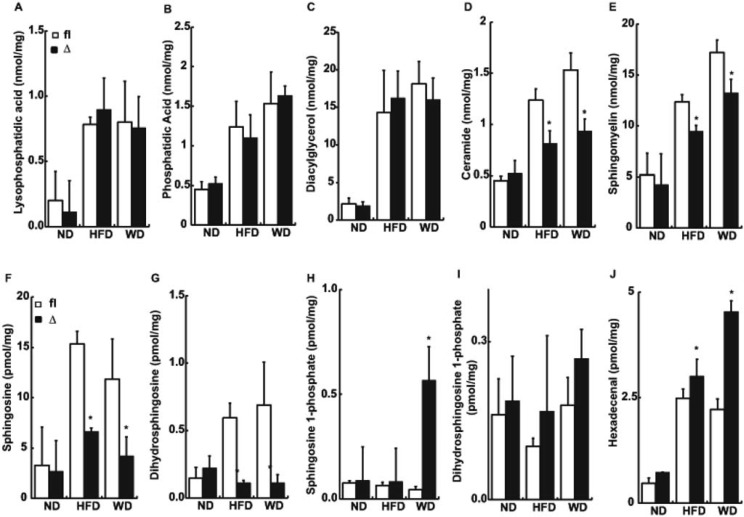
Effect of diet and LPP3 deficiency on adipose tissue lipid composition. Mice were fed normal chow, high fat diet (HFD), or Western diet (WD) for 8- weeks, at which time white adipose tissue was collected for measurement of the indicated lipids by HPLC electrospray ionization tandem mass spectrometry. (A) lysophosphatidic acid, (B) phosphatidic acid, (C) diacylglycerol, (D) ceramide, (E) sphingomyelin, (F) sphingosine, (G) dihydrosphingosine, (H) sphingosine 1- phosphate, (I) dihydrosphingosine 1- phosphate and (J) hexadecenal. Data for lysophosphatidic acid, phosphatidic acid, sphingomyelin, ceramide and diacylglycerol are totals of 12–15 abundant molecular species. Hexadecenal was quantitated as its semicarbazone derivative. Data shown are from *Plpp3*^*fl/fl*^ (fl/fl; open bars) and AP2-Cre/*Plpp3*^Δ^ (Δ; black bars) and presented as mean ± SD (n = 3 for ND and 6 for HFD and WD per group). *P < 0.01 (unpaired Student's t- test).

The LPP3 substrate PA is essential for triglyceride production, and its product DG is an intermediate in phospholipid synthesis. LPP3 can also dephosphorylate S1P and C1P which could potentially impact sphingolipid metabolism which has been linked to insulin resistance and inflammation. To investigate the potential role of LPP3 in regulating the lipid composition of adipose tissue, we measured levels of multiple common molecular species of glycero- and sphingo- phospholipids using HPLC ESI tandem mass spectrometry in lipid extracts of white adipose tissue from *Plpp3*^*fl/fl*^ (fl/fl) and AP2-Cre/*Plpp3*^Δ^ (Δ) mice fed normal chow, high fat diet or Western diet for 8 weeks. As reported by others [7], we observed diet-dependent increases in adipose tissue levels of LPA, PA and DG that presumably reflect the role of these lipids as active intermediates in triglyceride synthesis from dietary fats and carbohydrates. Levels of these glycerophospholipids and DG were not significantly different between *Plpp3*^*fl/fl*^ (fl/fl) and AP2-Cre/*Plpp3*^Δ^ (Δ) mice (Fig 3A, 3B and 3C). Diet- induced hyperlipidemia has also been associated with increased accumulation of sphingolipids in adipose [33] and other tissues [34]. In keeping with these reports, we observed diet-dependent increases in levels of ceramide and sphingomyelin in adipose tissue (Fig 3D and 3E). Interestingly, although still elevated in comparison to normal diet controls, the increases in adipose ceramide and sphingomyelin were blunted in LPP3 deficient AP2-Cre/*Plpp3*^Δ^ (Δ) mice in comparison to *Plpp3*^*fl/fl*^ (fl/fl) controls (Fig 3D, 3E and S3 Fig). Similarly, although present at considerably lower levels than ceramide and sphingomyelin, high fat or Western diet feeding also increased adipose levels of sphingosine and dihydrosphingosine. However, these increases were also blunted by LPP3 deficiency (Fig 3F and 3G). In contrast to the effects on these other sphingolipids, levels of S1P were unchanged by high fat or Western diet feeding in *Plpp3*^*fl/fl*^ (fl/fl) mice but increased in adipose tissue from LPP3 deficient AP2-Cre/*Plpp3*^Δ^ (Δ) mice on a Western diet (Fig 3H). No significant differences were observed in adipose dihydro-S1P levels under any of the conditions examined (Fig 3I). Finally, levels of hexadecenal which is the lipid product of S1P lyase catalyzed the degradation of S1P were also increased by high fat feeding and modestly augmented by LPP3 deficiency (Fig 3J). Adipose-deficiency of LPP3 had no effect on levels of these classes of lipids in liver (S4 Fig).

### LPP3 deficiency does not alter diet- induced obesity, yet improves insulin sensitivity in Western diet fed mice

**Fig 4:**
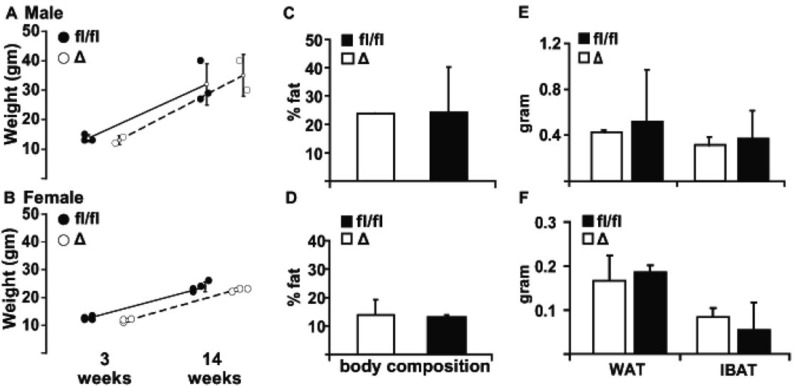
Deficiency of adipose LPP3 does not affect diet-induced obesity. Average body weight of male (A) and female (B) *Plpp3*^*fl/fl*^ (fl/fl; dark symbols) and AP2-Cre/*Plpp3*^Δ^ (Δ; open symbols) mice (n = 2–5/group) at 3 weeks and at 14 weeks of age following an 11-week high fat diet (HFD). The percentage of body fat determined by DEXA in male (C) and female (D) *Plpp3*^*fl/fl*^ (fl/fl; black bars) and AP2-Cre/*Plpp3*^Δ^ (Δ; open bars) mice at 20 weeks on HFD. The weight of white adipose tissue (WAT) and interscapular brown adipose tissue (IBAT) male (E) and female (F) *Plpp3*^*fl/fl*^ (fl/fl; black bars) and AP2-Cre/*Plpp3*^Δ^ (Δ; open bars) mice at 20 weeks on HFD. Results are presented as mean ± SD.

Having demonstrated a functional effect of adipose LPP3 deficiency on diet-dependent sphingolipid levels, we examined the impact on diet-induced adipose expansion. LPP3 deficiency had no effect on weight in either male (Fig 4A) or female (Fig 4B) fed a high fat diet starting at weaning. Specifically, in the cohort of animals fed Western diet for eight weeks, the absence of adipocyte LPP3 did not affect weight of either male (34.8 ± 3.8 versus 32.5 ± 3 g in *Plpp3*^*fl/fl*^ (fl/fl) and AP2-Cre/*Plpp3*^Δ^, respectively, P = 0.15) or female mice (26 ± 4.5 versus 25.7 ± 2.7 g in *Plpp3*^*fl/fl*^ (fl/fl) and AP2-Cre/*Plpp3*^Δ^, respectively, P = 0.86). The body composition analysis and body fat percentage, measured by DEXA, were similar in the *Plpp3*^*fl/fl*^ (fl/fl) and AP2-Cre/*Plpp3*^Δ^ (Δ) mice on high fat diet (Fig 4C and 4D). Finally, the weight of fat pads was also the same for both genotypes (Fig 4E and 4F). These results indicate that, unlike the soluble lipin1 PA phosphatase, deficiency of LPP3 does influence diet dependent expansion of adipose tissue.

**Fig 5:**
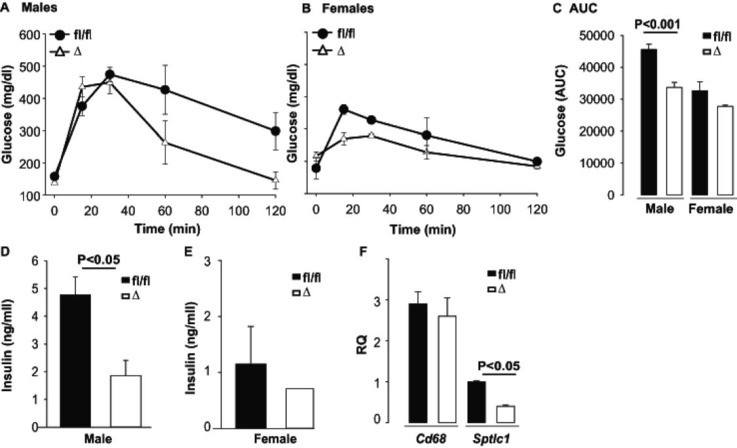
Adipose LPP3 deficiency improves glucose tolerance in mice fed Western diet. After a 5 hour fast, baseline glucose was measured in Western diet fed mice. The animals were then injected ip with glucose (2 g/kg body weight) in isotonic saline, and serial measurements of blood glucose levels were made at the indicated times up to 120 min. Blood glucose in male (A) and female (B) *Plpp3*^*fl/fl*^ (fl/fl; black symbols) and AP2-Cre/*Plpp3*^Δ^ (Δ; open symbols) mice are graphed as mean ± SD from 6 animals/group. The area under the curve (AUC; C) indicates a significant difference in male mice. Plasma insulin levels in *Plpp3*^*fl/fl*^ (fl/fl; black bars) and AP2-Cre/*Plpp3*^Δ^ (Δ; open bars) mice on Western diet were determined by ELISA and reported as mean ± SD for male mice (D) and female mice (E). Levels of expression of Cd68 and Sptlc1 in epididymal fat from *Plpp3*^*fl/fl*^ (fl/fl; dark symbols) and AP2-Cre/*Plpp3*^Δ^ (Δ; open symbols) mice on a Western diet (F). Values are reported relative to expression in fl/fl mice on normal chow. P -values were calculated using unpaired Student's t- test.

Accumulation of sphingolipids, particularly ceramides has been associated with insulin resistance in animal models and clinical studies [35]. We, therefore, determined the response to glucose challenge in mice with and without adipose LPP3. The Western diet fed mice were used for these experiments because these animals exhibited effects of LPP3 deficiency on diet-dependent sphingolipid levels. No differences were observed in baseline blood glucose or in blood glucose levels following i.p. glucose challenge in *Plpp3*^*fl/fl*^ (fl/fl) and AP2-Cre/*Plpp3*^Δ^ (Δ) mice fed normal chow (S1 Fig). Consistent with the development of glucose intolerance, blood glucose levels following i.p. glucose loading were higher in mice fed a Western diet for 8 weeks (peak glucose levels of 312 ± 34 versus 449 ± 34 mg/dl for males on normal and Western diet, respectively, P<0.01). In comparison to control fl/fl mice on Western Diet, a blunted response to i.p. glucose challenge occurred in male (Fig 5A) and to a lesser extent female (Fig 5B) AP2-Cre/*Plpp3*^Δ^ (Δ) mice on Western diet (Fig 5C). Plasma concentrations of insulin were also lower in male AP2-Cre/*Plpp3*^Δ^ (Δ) mice on Western diet (Fig 5D), and the lower levels persisted after glucose administration. In the first 30 min of glucose tolerance test, insulin levels were significantly lower in male AP2-Cre/*Plpp3*^Δ^ (Δ) mice on HFD than their litter fl/fl controls (area under the curve (AUC) 32 ± 4 versus 56 ± 3 ng/ml; P = 0.02; n = 5; S2 Fig). However, no difference in glucose levels for up to 120 minutes were observed following insulin injection (0.5U / kg) in fl/fl and AP2-Cre/*Plpp3*^Δ^ (Δ) mice on HFD (P = 0.832 for AUC in males and P = 0.728 for females; n = 8; S2 Fig). Gene expression analysis, performed by qualitative PCR, indicated that the absence of LPP3 did not alter expression of genes related to adipose differentiation such as *Adipoq* (not shown) or inflammation including *Cd68* (Fig 5F) and *Mcp1* (not shown) in epididymal white adipose tissue. Interestingly, mRNA levels for serine palmitoyl-CoA transferase (*Sptlc*), a key enzyme in sphingolipid biosynthesis, were lower in AP2-Cre/*Plpp3*^Δ^ (Δ) mice (Fig 5F) which may account at least in part for the decreased accumulation of ceramide and sphingomyelin we observed in these animals on high fat or Western diets.

## Discussion

Our results clearly show that LPP3 is dispensable for adipocyte development in mice and for triglyceride synthesis and storage in mice fed normal chow, high fat or Western diets. Although capable of dephosphorylating PA to generate the triglyceride precursor DG, LPP3 function in adipocytes is clearly distinct from the function of the soluble PA phosphatases encoded by the lipin genes. This distinction presumably reflects differences in physiological substrate preferences or substrate accessibility between these enzymes. LPPs including LPP3 may also function as regulators of the extracellular signaling actions of bioactive lysophospholipids, particularly LPA [36]. Evidence that LPA, its receptors and the LPA generating enzyme ATX are important regulators of adipocyte development and function comes from studies using cell culture systems and mice with inactivation of the ATX and LPA receptor genes [37]. Our studies suggest that LPP3 deficiency also does not impact these processes. Adipose tissue expresses all three LPP enzymes [14]. While the lack of an effect of LPP3 deficiency on adipocyte development and function could reflect redundancy with these enzymes, LPP3 function is non-redundant with LPP1 and LPP2. Most notably, disruption of *Plpp3* revealed an essential role for LPP3 in embryogenesis and vascular development that is not observed with disruption of *Plpp1/Ppap2a* or *Plpp2/Ppap2c* [10, 15–17, 38]. The phenotype observed with global deletion of *Plpp3* may relate in part to an essential role in endothelial cell biology, including barrier function and migration. Interestingly, the *Drosophila* LPP homolog Wunen is required for septate junction function in the trachea and to maintain blood-brain barrier [39]. In adipocytes, where junctions are not essential for tissue integrity or function, this apparently unique role for LPP3 may not be important. Alternatively, if dephosphorylation of extracellular bioactive lipid mediators is an important LPP function, then a non-cell autonomous role of LPP3 (expressed by stromal or other cells) may be sufficient to mediate biologically important effects of the enzyme on extracellular lipid metabolism and signaling in adipose tissue development and function.

In contrast to the lack of an effect of LPP3 deficiency on adipose development and triglyceride synthesis, we were surprised to observe that LPP3 deficiency blunted diet- dependent accumulation of ceramide and sphingosine in adipose tissue. Levels of sphingosine and dihydrosphingosine were also decreased in tissue obtained from LPP3 deficient high fat or Western diet fed animals. While we did not observe significant changes in adipose tissue dihydro- S1P levels with either LPP3 genotype or diet, S1P levels were significantly increased in LPP3 deficient adipose tissue from Western diet fed mice. Levels of the S1P lyase product, hexadecenal, were also elevated by high fat and Western diet feeding and these increases were significantly augmented by LPP3 deficiency. It is conceivable that LPP3 normally plays a role in the interconversion of S1P and dihydro-S1P with sphingosine and dihydrosphingosine. In the absence of LPP3, S1P accumulates and is irreversibly degraded by S1P lyase. While the steady- state levels of these sphingolipids are low relative to levels that accumulate with high fat or Western diet feeding, the flux of material through the sphingolipid synthesis pathway could be sufficiently high [40] to make accumulation and degradation of S1P and dihydroS1P a plausible mechanism for depletion of the precursor dihydrosphingosine. This depletion of dihydrosphingosine may contribute to the blunted diet-dependent accumulation of ceramide and sphingomyelin associated with adipose LPP3 deficiency. Alternatively, while a more comprehensive analysis of the impact of LPP3 deficiency on the sphingolipidome appears warranted, the decreased serine palmitoyl transferase expression observed in LPP3 deficient adipose tissue may also contribute to the blunted diet-dependent accumulation of adipose ceramide and sphingomyelin associated with adipose LPP3 deficiency. Our finding that adipose LPP3 deficiency is associated with improved glucose tolerance and insulin sensitivity in high fat and Western diet fed mice is consistent with other observations that these processes are impaired by the accumulation of these lipids, particularly ceramide, in adipose and other tissues [35, 41]. Interestingly, while LPP3 deficiency in vascular smooth muscle cells did not result in alterations in circulating S1P levels, inactivation of LPP3 in vascular endothelium or bone marrow derived cells resulted in changes in S1P signaling pathways that regulate lymphocyte trafficking [17]. Inactivation of LPP3 in neurons resulted in increased levels of S1P (but not other LPP3 substrates) in brain tissue which was also associated with defects in development and neuronal function that could be explained by alterations in S1P signaling [15]. Taken together, these observations and this report suggest that LPP3 may have unappreciated roles in regulation sphingolipid metabolism.

## Supporting information

S1 Fig Glucose tolerance in mice fed normal chow diet. After a 5 hour fast, baseline glucose was measured, and animals were injected ip with glucose (2 g/kg body weight) in isotonic saline. Serial measurements of blood glucose levels were made at the indicated times up to 120 min. Blood glucose in male (A) and female (B) *Plpp3*^*fl/fl*^ (fl/fl; black symbols) and AP2-Cre/*Plpp3*^Δ^ (Δ; open symbols) mice are graphed as mean ± SD from 6 animals/group. The mean area under the curve (AUC) is presented in C.
(PPTX)

S2 Fig Insulin levels after glucose or insulin administration in male mice. A. After a 5 hour fast, baseline insulin was measured, and male *Plpp3*^*fl/fl*^ (fl/fl; black bars) and AP2-Cre/*Plpp3*^Δ^ (Δ; open bars) were injected ip with glucose (2 g/kg body weight) in isotonic saline. Measurements of blood insulin levels (ng/ml) were made for up to 30 min and the area under the curve presented as mean ± SD. Results were compared by two tailed t-test. B. Insulin tolerance testing was performed and blood glucose at the indicated times in male *Plpp3*^*fl/fl*^ (fl/fl; black symbols) and AP2-Cre/*Plpp3*^Δ^ (Δ; open symbols) mice are graphed as mean ± SD from. The mean area under the curve (AUC) is presented in C. Results were compared by two-tailed t-test. 6–8 animals were used per condition.
(PPTX)

S3 Fig Effect of diet and LPP3 expression on adipose tissue lipid composition. Mice were fed high fat diet (HFD) for 8 weeks, at which time white adipose tissue was collected from *Plpp3*^*fl/fl*^ (fl/fl; black symbols) and AP2-Cre/*Plpp3*^Δ^ (Δ; open symbols) mice for measurement of the indicated lipids by HPLC electrospray ionization tandem mass spectrometry. The most abundant species of ceramides (A) and sphingomyelins (B) are presented as mean ± SD as described in Fig 3.
(PPTX)

S4 Fig No effect of adipose LPP3 expression on liver lipid composition. Mice were fed high fat diet (HFD) for 8 weeks, at which time liver was collected from *Plpp3*^*fl/fl*^ (fl/fl; black symbols) and AP2-Cre/*Plpp3*^Δ^ (Δ; open symbols) mice for measurement of the indicated lipids by HPLC electrospray ionization tandem mass spectrometry, as described in the text, and total lipid levels are presented as mean ± SD. SM = sphingomyelin; S1P = sphingosine-1-phosphate; DHS = dihydrosphingosine.
(PPTX)

## Abbreviations

ATX: Autotaxin
C1P: Ceramide-1-phosphate
DG: Diacylglycerol
DEXA: Dual-energy X-ray absorptiometry
IBAT: Interscapular brown adipose tissue
HPLC: High performance liquid chromatography
LPP: Lipid phosphate phosphatase
PA: Phosphatidic acid
LPA: Lysophosphatidic acid
MS: Mass spectrometry
ND: Normal diet
RQ: Relative quantification
S1P: Sphingosine 1-phosphate
SUB: Subcutaneous
HFD: High fat diet
WD: Western diet
WAT: White adipose tissue
