# Supplementary material for: Lipid phosphate phosphatase 3 regulates adipocyte sphingolipid synthesis, but not developmental adipogenesis or diet-induced obesity in mice
Source: PLoS One. 2018 Jun 11;13(6):e0198063. doi: 10.1371/journal.pone.0198063 (PMC5995365; doi:10.1371/journal.pone.0198063)
Supplement: S1 Fig — After a 5 hour fast, baseline glucose was measured, and animals were injected ip with glucose (2 g/kg body weight) in isotonic saline. Serial measurements of blood glucose levels were made at the indicated times up to 120 min. Blood glucose in male (A) and female (B) Plpp3fl/fl (fl/fl; black symbols) and AP2-Cre/Plpp3Δ (Δ; open symbols) mice are graphed as mean ± SD from 6 animals/group. The mean area under the curve (AUC) is presented in C. (PPTX) [file pone.0198063.s001.pptx]

## Slide 1
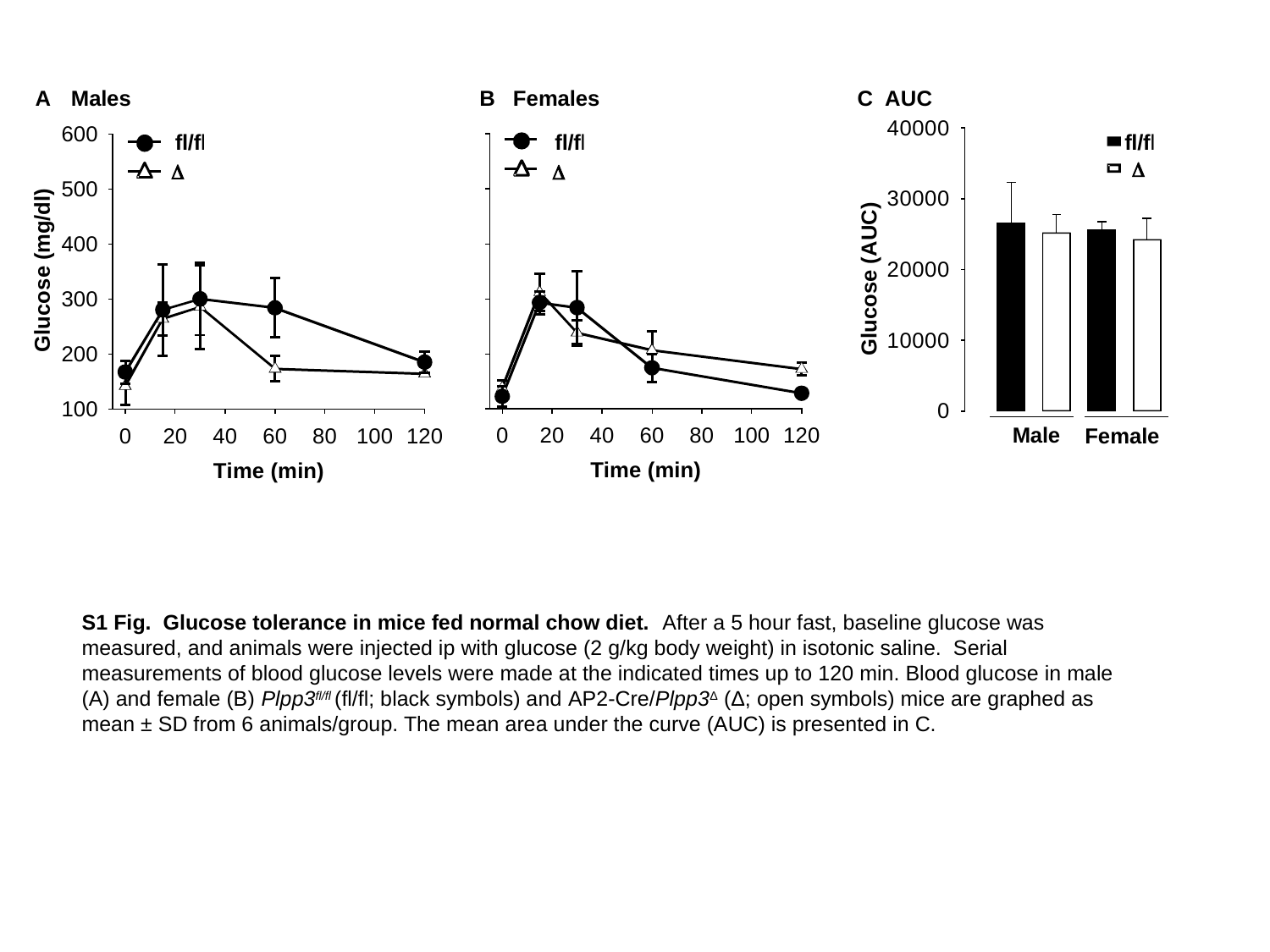

D
D
D
S1 Fig. Glucose tolerance in mice fed normal chow diet. After a 5 hour fast, baseline glucose was measured, and animals were injected ip with glucose (2 g/kg body weight) in isotonic saline. Serial measurements of blood glucose levels were made at the indicated times up to 120 min. Blood glucose in male (A) and female (B) Plpp3fl/fl (fl/fl; black symbols) and AP2-Cre/Plpp3Δ (Δ; open symbols) mice are graphed as mean ± SD from 6 animals/group. The mean area under the curve (AUC) is presented in C.
