# Supplementary material for: Lipid phosphate phosphatase 3 regulates adipocyte sphingolipid synthesis, but not developmental adipogenesis or diet-induced obesity in mice
Source: PLoS One. 2018 Jun 11;13(6):e0198063. doi: 10.1371/journal.pone.0198063 (PMC5995365; doi:10.1371/journal.pone.0198063)
Supplement: S2 Fig — A. After a 5 hour fast, baseline insulin was measured, and male Plpp3fl/fl (fl/fl; black bars) and AP2-Cre/Plpp3Δ (Δ; open bars) were injected ip with glucose (2 g/kg body weight) in isotonic saline. Measurements of blood insulin levels (ng/ml) were made for up to 30 min and the area under the curve presented as mean ± SD. Results were compared by two tailed t-test. B. Insulin tolerance testing was performed and blood glucose at the indicated times in male Plpp3fl/fl (fl/fl; black symbols) and AP2-Cre/Plpp3Δ (Δ; open symbols) mice are graphed as mean ± SD from. The mean area under the curve (AUC) is presented in C. Results were compared by two-tailed t-test. 6–8 animals were used per condition. (PPTX) [file pone.0198063.s002.pptx]

## Slide 1
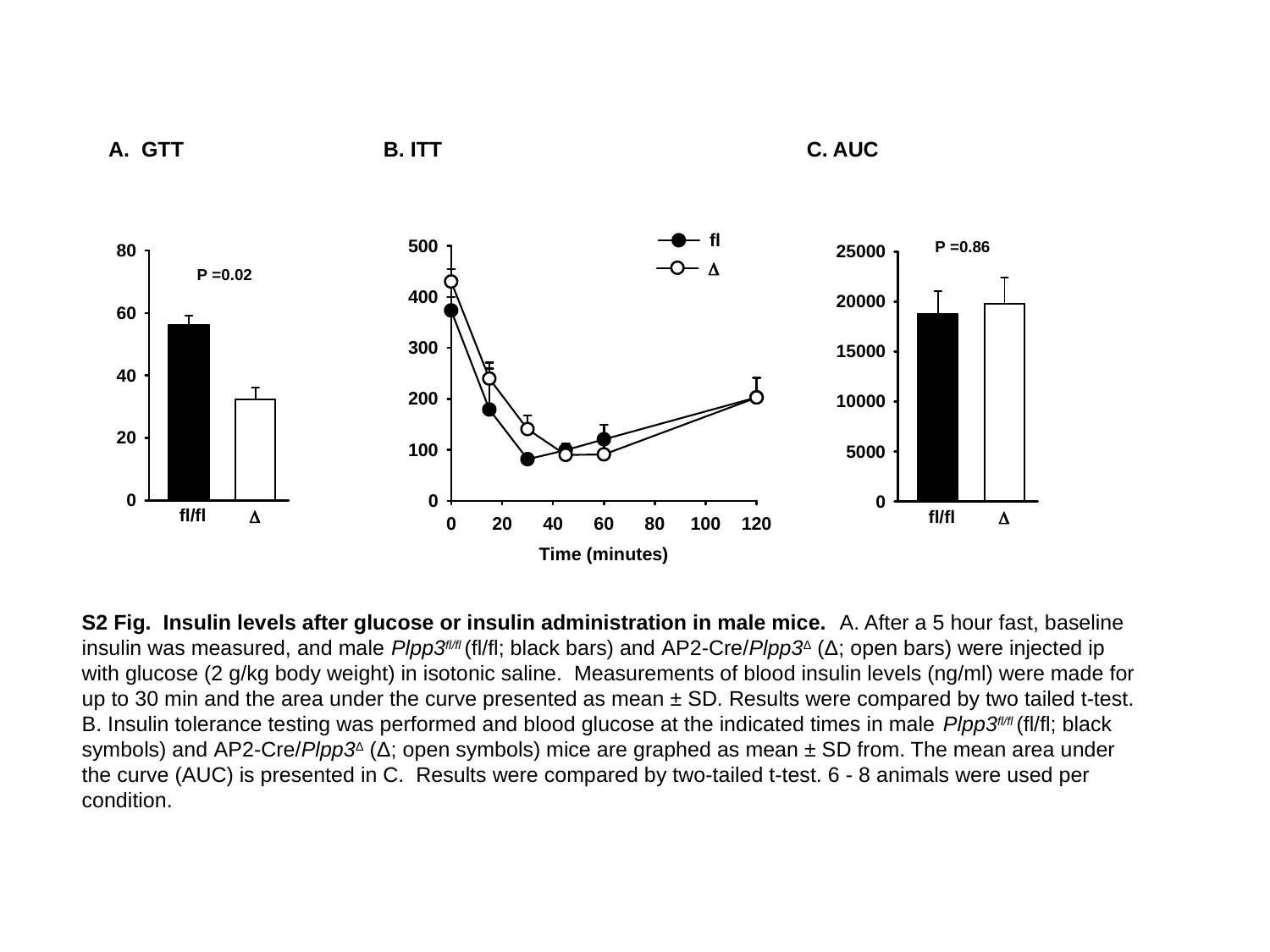

A. GTT
B. ITT
C. AUC
S2 Fig. Insulin levels after glucose or insulin administration in male mice. A. After a 5 hour fast, baseline insulin was measured, and male Plpp3fl/fl (fl/fl; black bars) and AP2-Cre/Plpp3Δ (Δ; open bars) were injected ip with glucose (2 g/kg body weight) in isotonic saline. Measurements of blood insulin levels (ng/ml) were made for up to 30 min and the area under the curve presented as mean ± SD. Results were compared by two tailed t-test. B. Insulin tolerance testing was performed and blood glucose at the indicated times in male Plpp3fl/fl (fl/fl; black symbols) and AP2-Cre/Plpp3Δ (Δ; open symbols) mice are graphed as mean ± SD from. The mean area under the curve (AUC) is presented in C. Results were compared by two-tailed t-test. 6 - 8 animals were used per condition.
