# Supplementary material for: Lipid phosphate phosphatase 3 regulates adipocyte sphingolipid synthesis, but not developmental adipogenesis or diet-induced obesity in mice
Source: PLoS One. 2018 Jun 11;13(6):e0198063. doi: 10.1371/journal.pone.0198063 (PMC5995365; doi:10.1371/journal.pone.0198063)
Supplement: S3 Fig — Mice were fed high fat diet (HFD) for 8 weeks, at which time white adipose tissue was collected from Plpp3fl/fl (fl/fl; black symbols) and AP2-Cre/Plpp3Δ (Δ; open symbols) mice for measurement of the indicated lipids by HPLC electrospray ionization tandem mass spectrometry. The most abundant species of ceramides (A) and sphingomyelins (B) are presented as mean ± SD as described in Fig 3. (PPTX) [file pone.0198063.s003.pptx]

## Slide 1
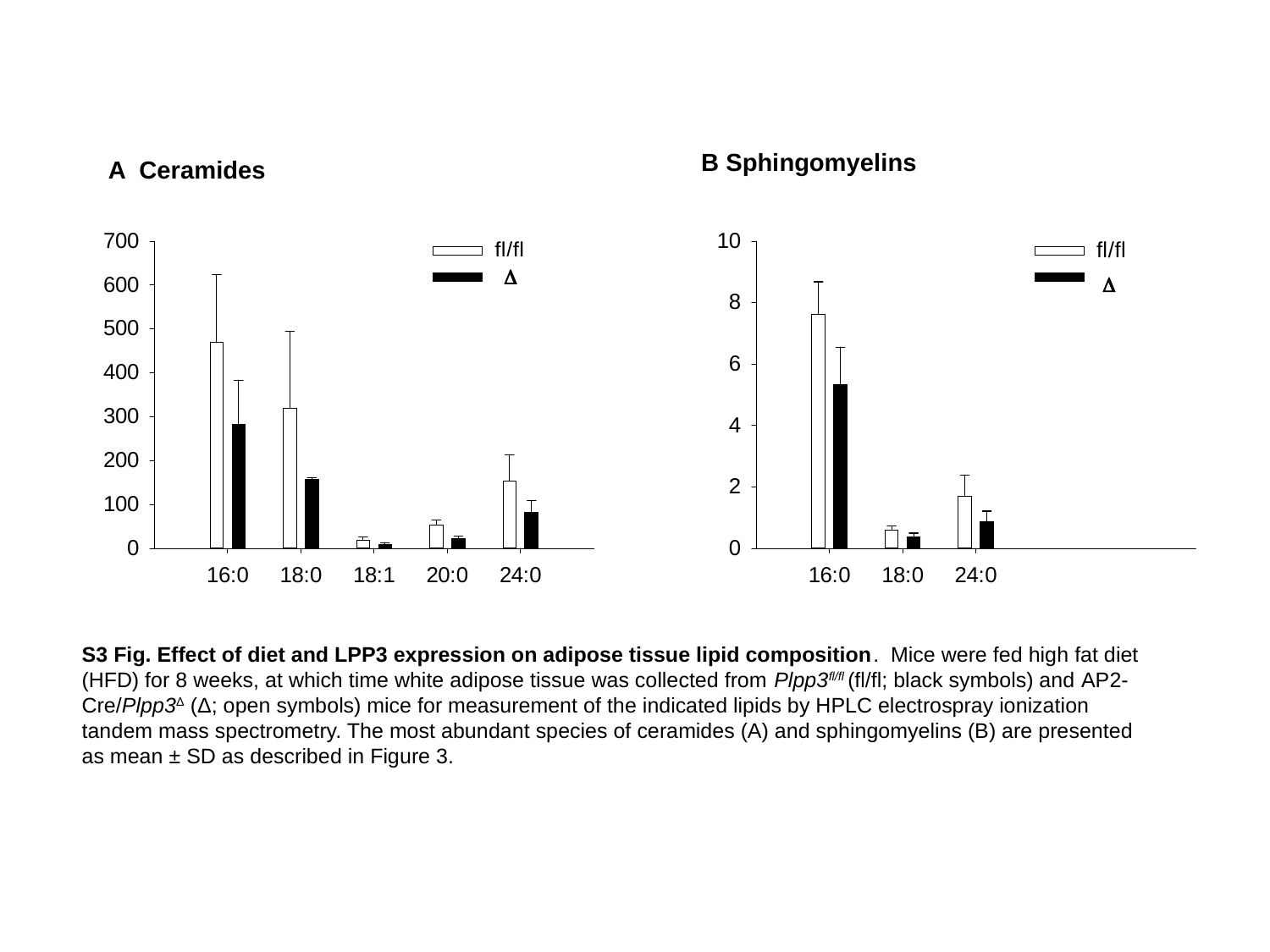

B Sphingomyelins
A Ceramides
D
D
S3 Fig. Effect of diet and LPP3 expression on adipose tissue lipid composition. Mice were fed high fat diet (HFD) for 8 weeks, at which time white adipose tissue was collected from Plpp3fl/fl (fl/fl; black symbols) and AP2-Cre/Plpp3Δ (Δ; open symbols) mice for measurement of the indicated lipids by HPLC electrospray ionization tandem mass spectrometry. The most abundant species of ceramides (A) and sphingomyelins (B) are presented as mean ± SD as described in Figure 3.
