# Supplementary material for: Lipid phosphate phosphatase 3 regulates adipocyte sphingolipid synthesis, but not developmental adipogenesis or diet-induced obesity in mice
Source: PLoS One. 2018 Jun 11;13(6):e0198063. doi: 10.1371/journal.pone.0198063 (PMC5995365; doi:10.1371/journal.pone.0198063)
Supplement: S4 Fig — Mice were fed high fat diet (HFD) for 8 weeks, at which time liver was collected from Plpp3fl/fl (fl/fl; black symbols) and AP2-Cre/Plpp3Δ (Δ; open symbols) mice for measurement of the indicated lipids by HPLC electrospray ionization tandem mass spectrometry, as described in the text, and total lipid levels are presented as mean ± SD. SM = sphingomyelin; S1P = sphingosine-1-phosphate; DHS = dihydrosphingosine. (PPTX) [file pone.0198063.s004.pptx]

## Slide 1
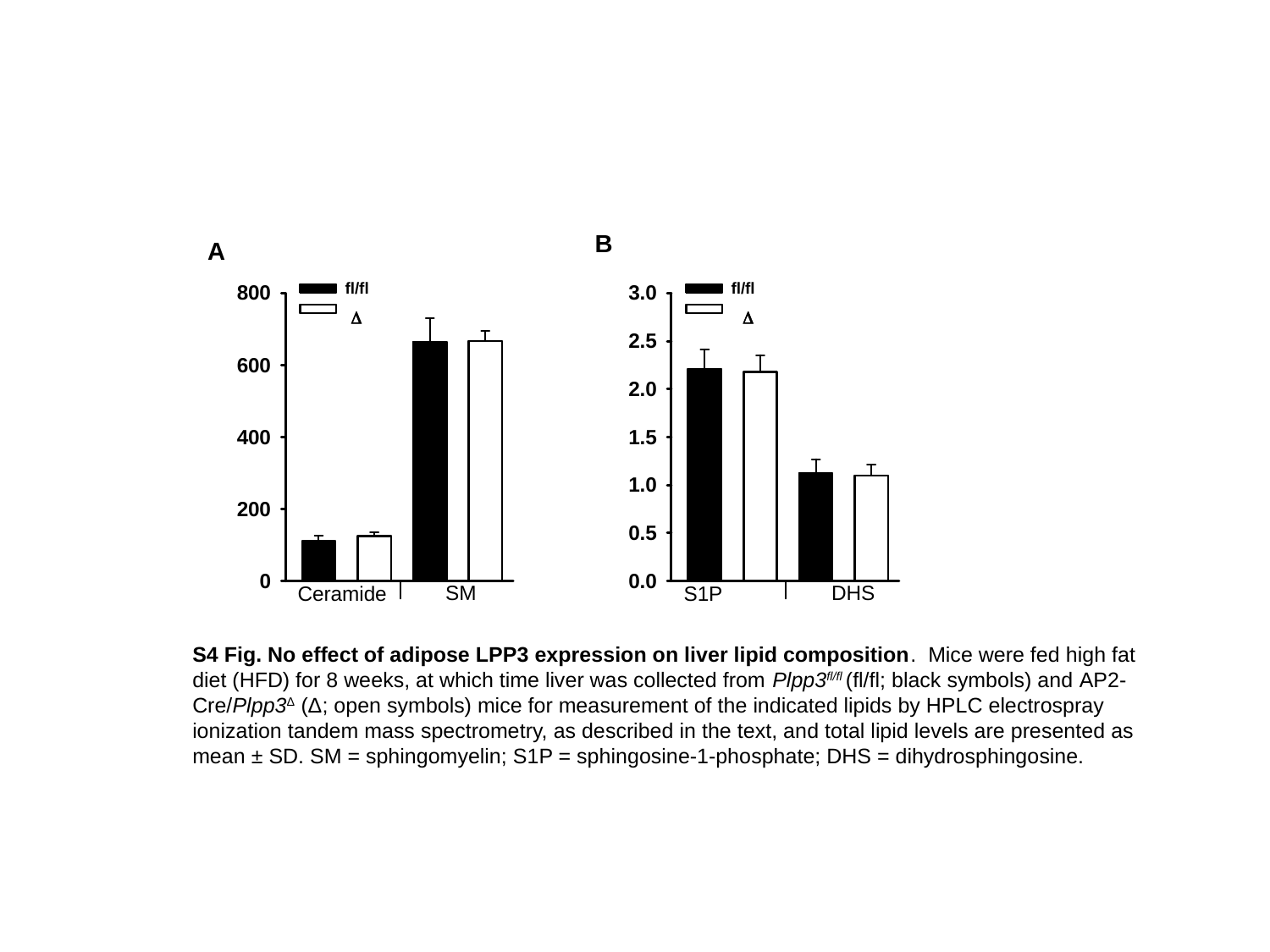

B
A
D
D
S4 Fig. No effect of adipose LPP3 expression on liver lipid composition. Mice were fed high fat diet (HFD) for 8 weeks, at which time liver was collected from Plpp3fl/fl (fl/fl; black symbols) and AP2-Cre/Plpp3Δ (Δ; open symbols) mice for measurement of the indicated lipids by HPLC electrospray ionization tandem mass spectrometry, as described in the text, and total lipid levels are presented as mean ± SD. SM = sphingomyelin; S1P = sphingosine-1-phosphate; DHS = dihydrosphingosine.
